# Mental health and substance use disorder comorbidities among Medicaid beneficiaries: Associations with opioid use disorder and prescription opioid misuse

**DOI:** 10.3934/publichealth.2023046

**Published:** 2023-08-10

**Authors:** James A. Swartz, Dana Franceschini, Kamryn Scamperle

**Affiliations:** 1 Jane Addams College of Social Work, University of Illinois Chicago; 2 University of Texas Health Science Center at Houston

**Keywords:** opioid use disorder, prescription opioid misuse, Medicaid, co-occurring substance use disorders, co-occurring mental health disorders, medical complexity, behavioral health comorbidities

## Abstract

**Background:**

Medicaid presently insures about one-fourth of the US population and disproportionately insures about 38 % of non-elderly adults with an opioid use disorder (OUD). Owing to Medicaid's prominent role insuring persons with an OUD and that Medicaid coverage includes pharmaceutical benefits, there has been considerable interest in studying potential prescription opioid misuse among Medicaid beneficiaries and identifying subpopulations at higher risk for misuse and possible progression to an OUD.

**Methods:**

The study goals were to explore the associations among prescription opioid misuse, OUD, and co-occurring mental health and other substance use disorders (SUD). We analyzed Illinois Medicaid 2018 claims data for 1102479 adult beneficiaries 18 to 64 years of age. Using algorithms based on previous studies, we first determined either the presence or absence of nine SUDS (including OUD), nine mental health disorders and likely prescription opioid misuse. Then, we subdivided the beneficiary sample into five groups: those who were prescribed opioids and evidenced either no, possible, or probable misuse; those evidencing an OUD; and those evidencing no opioid use or misuse.

**Results:**

Bivariate analyses, upset plots, and multinomial logistic regressions were used to compare the five subgroups on the prevalence of co-occurring SUDS and mental health disorders. Those with an OUD or with probable prescription opioid misuse had the highest prevalence of most co-occurring conditions with beneficiaries with an OUD the most likely to evidence co-occurring SUDS, particularly tobacco use disorder, whereas those with probable misuse had elevated prevalence rates of co-occurring mental health disorders comparable to those with an OUD.

**Conclusion:**

The medical complexity of persons with an OUD or misusing prescription opioids are considered in light of recent attempts to expand buprenorphine provision as a medication for OUD among Medicaid beneficiaries. Additionally, we consider the possibility of gender, co-occurring mental health disorders, and tobacco use disorder as important risk factors for progressing to prescription opioid misuse and an OUD.

## 1. Introduction

Currently, Medicaid insures about one-fourth of the US population or just over 81 million persons [1]. Designed primarily as a safety-net program to provide health insurance to the nation's poor and disabled, Medicaid beneficiaries are disproportionately from a racial/ethnic minority, have incomes either near or below the federal poverty level, and have disabling physical and mental health conditions, including substance use disorders (SUD) [2]. Medicaid plays an even more significant role with respect to SUD treatment and is the largest public payer of SUD services in the US [3]. Current estimates are that Medicaid insures about 38 % of non-elderly adults with an opioid use disorder (OUD) overall, with the OUD prevalence rate higher among beneficiaries enrolled under the Affordable Care Act (ACA) expansion provisions [4],[5].

Because Medicaid insures such a large proportion of persons with an OUD, when the U.S. Congress acted to address the decades-long and ongoing opioid epidemic in 2018, they passed legislation that specifically targeted the provision of OUD treatment funded by Medicaid via the Substance Use Disorder Prevention that Promotes Opioid Recovery and Treatment for Patients and Communities (SUPPORT) Act (Public Law 115–271) [5]. The SUPPORT Act aims to expand and improve OUD treatment services for Medicaid beneficiaries, thereby reducing opioid-related overdoses and associated fatalities and improving health outcomes generally.

As one of 15 states receiving funding under the SUPPORT Act, Illinois undertook a series of related studies to determine the prevalence of OUD among Medicaid beneficiaries and to identify treatment barriers that could be addressed to expand and improve OUD treatment. Subsequently, several studies were conducted to assess these issues among state Medicaid beneficiaries and to inform the state's efforts to expand OUD treatment availability and uptake. This paper presents the findings of one of those studies, which sought to determine the prevalence of prescription opioid misuse and associated psychiatric and substance use comorbidities. Comprehensive and detailed information on the background, procedures, and findings for this and all other studies conducted under the Illinois SUPPORT Act initiative is available in the project final report [6].

## 2. Background

One of the more common and well-documented pathways by which many individuals have become addicted to opioids is primarily through the use of prescription opioids for analgesic purposes [7],[8]. The beginning of the now decades-long opioid epidemic in the US occurred in the 1990s, when pharmaceutical companies began aggressively promoting opioids to treat pain, while falsely claiming the formulations used minimized or even precluded the possibility of opioid dependence [7],[9],[10]. As a result of the pharmaceutical industry's persuasive lobbying and advertising campaigns, opioid analgesics were prescribed more liberally, causing many people to misuse them. This included taking more than the prescribed amount, using them without a prescription, or using them for their psychological effects rather than their pain-relieving properties. Such misuse put these individuals at risk of developing a dependency on these drugs.

Because the cost of prescription opioids can be much higher than the cost of illegally manufactured and distributed drugs such as heroin, many persons who either misused or became addicted to prescription opioids have an increased risk of transitioning to purchasing opioids illegally in various forms, including heroin and illicitly manufactured or diverted prescription opioids [11]. In 2016, the CDC enacted strict guidelines on prescribing opioids, with the intention of reversing the effects of overprescribing in the prior decades. However, the guidelines resulted in unintended consequences such as under-treatment of persons with chronic pain and too rapid tapering off of prescribed opioids, potentially hastening the transition to illegally purchased opioids [12],[13]. Research has found that the stricter guidelines resulted in increased overdoses and ED-related visits for opioid poisoning. These adverse consequences led the CDC to publish a revision in 2022, which advocated for a more nuanced, patient-centered approach to prescribing opioids for pain management [14],[15]. The transition to and/or use of illegally manufactured opioids sold on the street now underlies the majority of opioid overdose-related fatalities, which have continued to increase through 2021 [9],[16]. Much of that increase has been due to the use of potent synthetic opioids such as illegally manufactured fentanyl either in an admixture or completely supplanting heroin, further increasing opioid-related overdoses and fatalities [17]–[19].

Owing to Medicaid's prominent role insuring persons with an OUD/SUD and the fact that Medicaid coverage includes pharmaceutical benefits, there has been considerable interest in studying potential misuse among Medicaid beneficiaries receiving opioid prescriptions for noncancer-related pain and identifying subpopulations at a higher risk for misuse [20]–[26]. Research demonstrating the associations among co-occurring psychiatric and SUDs and the risk for an OUD is most relevant to the present study. For example, a recent systematic review on the co-occurrence of OUD with psychiatric disorders concluded that multiple studies had identified elevated rates of multiple psychiatric disorders such as depression, anxiety, attention deficit hyperactivity disorder (ADHD), post-traumatic stress disorder (PTSD), bipolar disorder, and anti-social personality disorder among persons also diagnosed with an OUD [27],[28]. An investigation of a potential genetic predisposition toward major depressive disorder, anxiety and stress-related disorders, and increased prescription opioid use found evidence of a genetic liability common to both psychiatric disorders as well as prescription opioid use [29]. Similarly, other recent research has found that co-occurring SUDs, most prominently alcohol use disorder as well as misuse of benzodiazepines, cocaine, and stimulants, are common among persons presenting for treatment with an OUD [30],[31].

Much, if not most, of the research on co-occurring psychiatric and other SUDs has focused on OUD. To the best of our knowledge, there has been relatively fewer studies on the extent of co-occurring behavioral health conditions among persons receiving prescription opioids and whether those with co-occurring conditions are more likely to misuse opioid analgesics as a precursor to developing an OUD, though some related work exists.

Using national data, Davis et al. found that the 16% of Americans with a mental health disorder received just over half (51.4%) of all opioid prescriptions in a calendar year [32]. A study of Iraq and Afghanistan veterans with a mental health diagnoses showed a higher prevalence of adverse clinical outcomes including opioid accidents and overdoses [33]. More recently and most germane to the present study, research performed by Jennings et al. [34] used electronic health record data to examine the co-occurrence of psychiatric, substance use, and medical disorders among three groups with varying levels of opioid exposure that ranged from no opioid prescriptions to chronic exposure, defined as having received 10 or more prescriptions in a 12-month period. The study included an additional comparison group consisting of those who had been diagnosed with an OUD. Among the main findings were higher rates of alcohol, tobacco, and cannabis use disorders among persons in the OUD group compared to the other groups, with prevalence rates generally increasing with prescription opioid exposure. Rates of co-occurring psychiatric disorders, most prominently bipolar disorder, anxiety disorders, and depression, were also the most elevated among those in the chronic exposure or OUD groups.

Our goals for this study were to further explore the associations among prescription opioid analgesic misuse and co-occurring mental health and SUDs. Using a different but similar classification system for characterizing the likely degree of prescription opioid misuse used in the Jennings et al. study, we compared the co-occurring condition prevalence for a larger number of SUDs and mental health disorders. We examined co-occurring conditions using multivariable multinomial logistic regressions to assess the relative risk for each SUD and mental health disorder among the prescription opioid misuse groups and persons with an OUD. Given the prominence Medicaid plays in insuring persons with OUDs as described above, we focused our study on the Illinois adult Medicaid population.

## 3. Materials and methods

### 3.1. Setting

This study was conducted as one of a series of studies to assess the prevalence of opioid use and misuse among adults insured under a Medicaid managed care plan, which insures approximately 80% of all Illinois Medicaid beneficiaries. Funding was provided by the Illinois Department of Healthcare and Family Services through a grant from the Centers for Medicare and Medicaid Services (CMS). The University of Illinois Chicago (UIC) IRB reviewed the study protocol and granted an exemption, as the analysis of de-identified Medicaid claims data was determined to not constitute human subjects research. Because the study was a secondary analysis of de-identified claims data, no informed consent was obtained.

### 3.2. Sample

Illinois claims data for adults enrolled in Medicaid between January 2018 to December 2018 were obtained from CMS through a request to the Research Data Access Center (ResDAC) at the University of Minnesota. CMS provided the Illinois claims data as a Transformed Medicaid Statistical System (T-MSIS) Analytic Files (TAF) extract. TAF extracts optimize the claims data reported by the states to CMS for use in research studies. The files provided included the following: demographic and enrollment information and claims related to inpatient hospital services; long-term care services; other and professional services; and pharmacy claims. The extract files included enrollment and associated claims data for Illinois Medicaid/CHIP beneficiaries 18 years of age or older who were enrolled in Medicaid at least one day in 2018 (N = 1489535).

For the purposes of the analyses, we further excluded persons insured under a fee-for-service program, those 65 or older who were dually eligible for Medicaid and Medicare, and those who lived outside of Illinois at their last determination. Moreover, we excluded persons who were not enrolled for at least 300 days or who had more than a 45-day gap in coverage over the course of the year. The intent of these criteria was to assess only those individuals who had been consistently insured by Medicaid and for a long enough period of time to accumulate a record of treatment services provided for an OUD/SUD and/or evidence prescription opioid misuse [35]. Application of these exclusion criteria resulted in a final analytic sample of 1102479 Illinois Medicaid beneficiaries.

### 3.3. Measures

#### 3.3.1. Prescription opioid misuse and opioid use disorder

The primary independent variable was a 5-category measure based on the following categories: 1) no prescription opioid use or misuse and no detected OUD; 2) prescription opioid use but no misuse detected; 3) prescription opioid use with possible misuse detected; 4) prescription opioid misuse with probable misuse detected; and 5) diagnosis of and/or treatment for an OUD.

The first category, no prescription opioid use or misuse or OUD, was assigned when a beneficiary did not meet criteria for any other category. To estimate the risk for prescription opioid misuse, we used an algorithm developed by Donohue et al. and validated it in a subsequent study using Pennsylvania Medicaid claims data [24],[36]. Briefly, beneficiaries receiving any opioid analgesic prescriptions were classified into one of three misuse categories — no misuse, possible misuse, or probable misuse — based on three criteria: 1) number of opioid prescribers within the year; 2) number of pharmacies where opioid prescriptions were filled within the year; and 3) days of supplied long-and short-acting opioids. As described by Cochran et al., the 3-category misuse indicator is based on the following coding scheme and score assignment: number of opioid prescribers (≤2 prescribers = 0; 3–4 prescribers = 1; ≥5 prescribers = 2); number of pharmacies used (≤2 = 0; 3–4 pharmacies = 1; ≥5 pharmacies = 1); days supplied of short-acting opioids and days of supply of long-acting opioids (≤185 days = 0; 186–240 days = 1; >240 days = 2). Scores on each measure for each beneficiary were totaled and a misuse category assigned: no misuse (0–1); possible misuse (2–4); and probable misuse (≥5) [24].

We excluded beneficiaries from the misuse classification who were not prescribed an opioid analgesic, those identified as having an OUD or being treated for cancer, persons in long-term or hospice care, and those identified as receiving a prescription for medication for treating an OUD (MOUD) (e.g., receiving methadone or suboxone). We identified opioid prescriptions by matching pharmacy claims with the national drug code numbers (NDCs) published in the CDC's file listing NDCs for opioid analgesics [37].

In the Illinois pharmacy claims file, we identified 124814 Illinois beneficiaries (7.5% of the total sample) who received at least one prescription for an opioid analgesic in 2018. Of these, the majority (82814 or 66.7%) evidenced no misuse based on their prescription pattern. However, the analyses also identified 36795 (29.6% of beneficiaries with an opioid analgesic prescription) with possible misuse and 4644 (3.7% of beneficiaries with an opioid analgesic prescription) evidencing probable misuse. Expressed as percentages of the total analytic sample of 1102479, there was possible prescription opioid misuse among 3.3% of 2018 Medicaid beneficiaries and probable misuse among 0.4% of beneficiaries, for an overall estimate of 3.7% possible or probable prescription opioid misuse.

To assess the validity of the prescription opioid misuse classification, we conducted analyses modeled after comparable validity analyses run by Cochran et al. on Pennsylvania Medicaid beneficiary data [24]. The three groups – no, possible, and probable prescription opioid misuse – were compared on percentages experiencing an ED visit or hospitalization in the past-year for an opioid-related overdose. Compared to beneficiaries with no discernible prescription opioid misuse based on their opioid analgesic prescription pattern, those with possible prescription opioid misuse had over twice the risk of an ED visit or hospitalization related to an overdose (relative risk ratio = 2.4; 95% confidence interval = 2.0–2.9). Those with probable prescription opioid misuse had almost five times the risk of an opioid overdose-related ED visit or hospitalization (relative risk ratio = 4.8; 95% confidence interval = 3.4–6.8). Based on these results, as applied to the 2018 Illinois Medicaid prescription claims data, we concluded the misuse classification algorithm had predictive validity and could identify persons with possible or probably prescription opioid misuse who are at elevated risk for an overdose that would require an ED visit or hospitalization.

To determine the presence (or absence) of an OUD among Illinois Medicaid beneficiaries, we used treatment algorithms detailed in the *Technical Specifications Code Book* that were developed to provide Congress with state-by-state and national estimates of the SUD treatment services received by Medicaid and CHIP beneficiaries [35]. The coding algorithms analyze the TAF claims data for the diagnosis or treatment of an OUD based on the types of services received and the ICD-10 diagnostic or procedural codes. A list of the qualifying diagnostic and procedural codes for determining an OUD or opioid-related overdose were obtained from the CMS Chronic Conditions Warehouse (CCW) algorithms for other chronic health, mental health, and potentially disabling conditions [38].

#### 3.3.2. Mental health and substance use disorders

The CCW diagnostic and procedure codes specified in the published algorithms were also used to assess for the presence of and treatment for mental health disorders and SUDs other than an OUD [38]. We used the provided algorithms to determine the presence or absence of the following classes of mental health disorders: anxiety, depression, bipolar disorder, schizophrenia/psychotic disorder, PTSD, personality disorder, ADHD, autism, and an intellectual disability. The SUDs assessed included disorders associated with the following drugs: alcohol, cannabis, cocaine, other stimulants, hallucinogens, tobacco, and inhalants.

#### 3.3.3. Demographics

We obtained the following demographic information from the TAF demographics and enrollment file: gender (male or female); race/ethnicity (Non-Hispanic White, Non-Hispanic Black, Hispanic, or other); age group (18–25 years, 26–35 years, 36–45 years, 46–54 years, and 55–64 years); marital status (legally married, legally separated, divorced, separated, widower, and never married/partnered); veteran (yes/no); US citizen (yes/no); and deceased (yes/no). We included these variables as covariates in the multinomial logistic regression models assessing the probabilities of co-occurring conditions by opioid use category to control for any effects of demographic differences among the opioid use groups.

### 3.4. Analyses

All analyses were conducted using either Stata v.17.1 or R (version 4.1.3) statistical software [39],[40]. Data were first screened to identify missing and out-of-range values, after which we ran bivariate descriptive analyses of the demographic data, co-occurring SUDs, and co-occurring mental health disorders disaggregated by the opioid use/misuse groups. Then, we ran a second set of descriptive analyses showing the bivariate associations among the five opioid use/misuse categories and each of the eight SUDS and 10 mental health disorders assessed. Moreover, we calculated the total number of co-occurring SUDS (excluding OUD) and mental health disorders and compared these by opioid use/misuse group. Significance levels for all bivariate descriptive models with categorical variables were assessed using Pearson's Chi-Square statistic, with effect size estimates obtained using Cramer's V [41]. Significance testing for interval level measures, age in years, and numbers of co-occurring SUDs and mental health disorders were performed using bivariate regression models estimated with robust standard errors and effect size assessed using R-square.

To determine which conditions most frequently co-occurred within the same year either alone or in combination with opioid use, we ran a series of upset plots, which display the frequency of each disorder individually and in combination with other disorders. We used the R package “Complex Upsets” to generate the upset plots [40],[42]. For these analyses, we combined the probable and possible prescription opioid misuse groups into a single group, as well as combined the no prescription opioid misuse and no opioid use groups into one group.

Next, we ran a series of multinomial logistic regressions, one for each mental health and substance use disorder, to assess for differences among the five opioid use categories controlling for demographic differences. The reference category for all multinomial logistic models consisted of Medicaid beneficiaries with no detected opioid use in the past year. Each model controlled for the following sociodemographic covariates: gender, race/ethnicity, age in years, marital status, veteran status, and citizenship status. Results of these comparisons are reported as relative risk ratios for each group in relation to the no-opioid-use reference group with statistical significance assessed by a z-test of the regression coefficient obtained for each disorder.

Preliminary analyses indicated two of the demographic covariates, race/ethnicity (38880 or 3.5% of the sample) and marital status (137727 or 12.5% of the sample), had relatively large amounts of missing data. We ran a sensitivity analysis using multiple imputations through chained equations to check if missing data on these covariates affected the relative risk ratio estimates and confidence intervals. The number of imputations was set at 15 based on results from the Stata add-on program “how_many_imputations,” which assesses the number of imputations needed to obtain stable point and standard error estimates [43]. Then, we reran the multinomial logistic models on the imputed data and compared the relative risk ratios and 95% confidence intervals to the corresponding estimates based on the non-imputed data. Because both sets of analyses produced comparable results that did not have an appreciable effect on either the obtained estimates or their substantive interpretations, we present the multinomial logistic regression results based on the non-imputed data but provide results based on the imputed data in the Supplementary.

## 4. Results

### 4.1. Demographic comparisons

Demographic results disaggregated by OUD and prescription opioid misuse status are shown in Table 1. Overall, the sample of adult Illinois Medicaid beneficiaries was composed predominantly of women (60.0%), non-Hispanic White (45.5%) or non-Hispanic Black (36.1%), between the ages of 26 to 45 years with a mean age of 39.0 years, those who had never been married or living with a partner (61.9%) and were U.S. citizens (96.1%). Less than one percent were veterans and 0.1% of individuals became deceased over the course of the year.

Although the demographic comparisons of the five opioid use/misuse groups were significant except for veteran status, the effect size estimates, which account for the large sample size, indicate the size of these differences was relatively small. Values for Cramer's V ranged from 0.01 to 0.07 for all variables except veteran status and the deceased group, which were 0.0, indicating only weak associations and no association, respectively. Interestingly, women were more likely than men to evidence possible (71.8% *vs*. 28.2%) or probable opioid misuse (70.1% *vs*. 29.9%), whereas men were more likely to have an OUD (54.8% *vs*. 45.2%). The largest racial/ethnic proportion of participants in each group were non-Hispanic Whites; however, the proportional difference between racial/ethnic groups was smallest among those with an OUD (49.4% NH-White *vs*. 41.6% NH-Black). Persons misusing prescription opioids or with an OUD were about four to five years older than those with no opioid use or no indication of misuse. Those with no detected opioid use and no misuse were also more likely to be married (19.0% no opioid use, 19.6% no misuse) compared with those with probable opioid misuse (12.9%) and especially those with an OUD (7.1%). Those with probable misuse (mean age = 45.6) and possible misuse (mean age = 44.8) tended to be older compared to those with no detected misuse (mean = 40.6). Although the absolute numbers and percentages were small, those with probable misuse (1.0%) and an OUD (0.7%) were more likely to have become deceased in 2018 than those with no detected misuse (0.2%) or those who did not use opioids (0.1%).

### 4.2. Co-occurring conditions

The top half of Table 2 shows the bivariate results for co-occurring SUDS by OUD use/misuse group. As can be seen from an inspection of the percentages for each co-occurring SUD overall and within opioid use/misuse group, there were several strong associations. In particular, nicotine use disorder was especially common among persons with an OUD (40.4%) and persons identified as probable misusers of prescription opioids (31.8%). Alcohol use disorder was most frequently detected as co-occurring among persons with an OUD (18.7%), as was cocaine use disorder (15.7%) and cannabis use disorder (11.7%). The probable prescription opioids misuse group had the next highest prevalence of these same disorders, followed by those in the possible misuse category. Medicaid beneficiaries with no misuse or no detected use of opioids had the lowest prevalence for all of the assessed SUDs. On average, a beneficiary with an OUD had at least one other co-occurring SUD (mean = 1.1, *SD* = 1.4); the bivariate OLS regression indicated that opioid use category accounted for 11% of the variance in a number of co-occurring SUDs.

The findings for co-occurring mental health disorders are shown in the bottom half of Table 2. Although the overall pattern of results obtained for co-occurring mental health disorders is similar to those for SUDs — persons with no or only possible opioid misuse having the lowest prevalence of the assessed mental health disorders — there was a different pattern for those with an OUD and those with probable misuse of prescription opioids. Although beneficiaries with an OUD had a higher prevalence of co-occurring SUDs, for three of the four most common co-occurring mental health disorders, those with probable prescription opioid misuse had a higher or near equivalent prevalence than beneficiaries with an OUD: anxiety disorders (42.4% probable misuse *vs*. 34.3% OUD); depression (33.6% *vs*. 32.8%); and bipolar disorder (18.8% *vs*. 19.0%). Additionally, those with probable prescription opioid misuse had a comparable number of co-occurring mental health conditions (mean = 1.2, *SD* = 1.3) compared with those with an OUD (mean = 1.1, *SD* = 1.4). Finally, though the association between the number of co-occurring mental health disorders and opioid use category was lower (*R*-square = 0.07) than for the co-occurring SUDs (*R*-square = 0.11), it was still statistically significant.

### 4.3. Disorder Combinations

**Figure 1.**
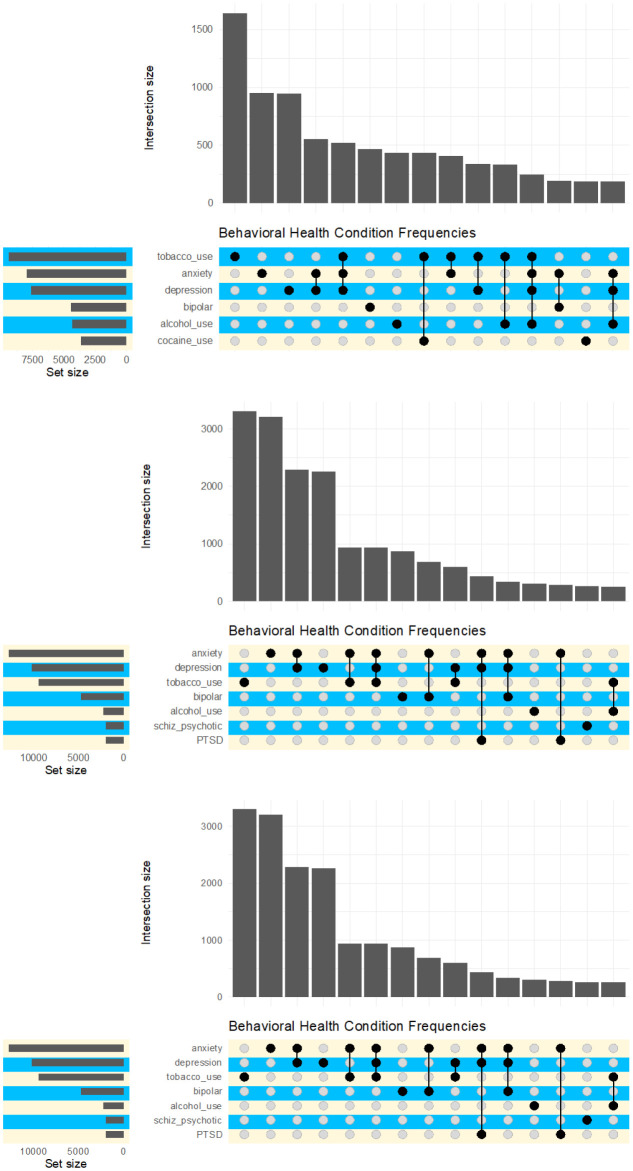
Co-occurring Substance Use and Mental Health Disorder Combinations by Opioid Use Group. **Note:** Results based on all adult Illinois residents ages 18 to 64 years of age enrolled in a Medicaid/CHIP managed care program. Graphs show the 15 most frequent combinations of co-occurring conditions for each opioid use group. The snall graph on the left side of each chart – set size - indicates the frequency of each substance across combinations, ordered from most to least frequent. The large bar chart-intersection size-indicates the frequency of each combination of substances and mental health conditions.

The three graphs in Figure 1 show the 15 most common combinations of co-occurring SUD or mental health disorders for beneficiaries with an opioid use disorder, probable or possible opioid prescription misuse, and no opioid use or misuse respectively. Among all opioid use categories, tobacco use disorder, anxiety or depression disorders, and bipolar disorders were the most frequently co-occurring conditions, either alone or in combination with other disorders. For beneficiaries treated for an OUD, tobacco use disorder was by far the most common co-occurring condition followed by anxiety, depression, and bipolar disorder. Moreover, tobacco use disorder frequently co-occurred with multiple other health conditions. Alcohol and cocaine use disorders were the other most frequently co-occurring SUDs among persons with an OUD.

For beneficiaries evidencing probable or possible prescription opioid misuse, tobacco use disorder was also the most common co-occurring condition when it was the only other condition besides prescription misuse. However, anxiety and depression occurred more frequently overall, as these disorders tended to occur more often in combination with other disorders than tobacco use disorder. Schizophrenia/psychotic disorder and PTSD were also among the most common co-occurring condition combinations with prescription opioid misuse, though these disorders occurred less often among persons with an OUD.

The bottom graph in Figure 1 indicates that anxiety, depression, and tobacco use disorder were also the most common disorders among beneficiaries where no opioid use or misuse was detected. Unique among the frequently occurring conditions for this group was cannabis use disorder, which did not co-occur with any other disorder.

**Table 1.** Demographics by opioid use category.

**Project**	**Prescription opioid misuse category**							
	**No detected opioid use (*N* = 954903)**	**No misuse (*N* = 82814)**	**Possible misuse (*N* = 36795)**	**Probable misuse (*N* = 4644)**	**Opioid use disorder (*N* = 23323)**	**Total (*N* = 1102479)**	**Sig**	**Cramer's V/R-squared**
**Sex**							***	0.07
Female	59.4	64.7	71.8	70.1	45.2	60.0		
Male	40.6	35.3	28.2	29.9	54.8	40.0		
**Race/ethnicity**							***	0.05
Non-Hispanic White	44.2	53.3	58.6	55.1	49.4	45.5		
Non-Hispanic Black	36.4	34.0	30.7	36.6	41.6	36.1		
Hispanic	15.2	10.5	9.0	7.1	8.3	14.4		
Other	4.3	2.3	1.6	1.2	0.8	3.9		
**Age group (in years)**							***	0.06
18–25	19.3	12.0	6.4	4.4	5.0	18.0		
26–35	28.2	28.3	19.9	18.9	23.6	27.8		
36–45	20.6	23.2	22.4	23.2	21.9	20.9		
46–54	15.1	18.0	24.3	24.7	25.6	15.9		
55–64	16.7	18.5	27.0	28.8	23.9	17.4		
**Age in years (mean/*SD*)**	38.5 (13.1)	40.6 (12.5)	44.8 (12.0)	45.6 (11.6)	44.1 (11.7)	39.0 (13.1)	***	0.01
**Marital status**							***	0.04
Legally married	19.0	19.6	17.5	12.9	7.1	18.4		
Legally separated	0.7	1.0	1.4	1.4	0.9	1.2		
Divorced	8.6	11.9	16.1	17.2	10.6	13.3		
Separated	2.3	3.2	3.9	3.7	2.8	3.5		
Widowed	1.3	1.5	2.1	1.9	1.6	1.7		
Never married/partnered	68.2	63.2	59.1	63.0	77.1	61.9		
**Veteran**	0.6	0.6	0.5	0.7	0.5	0.6	NS	0.00
**US citizen**	95.8	97.5	98.4	98.5	99.2	96.1	***	0.04
**Deceased**	0.1	0.2	0.4	1.0	0.7	0.1	***	0.0

Note: (1) Results based on all adult Illinois residents ages 18 to 64 years of age enrolled in a Medicaid/CHIP managed care program in calendar year 2018 for 300 or more days with no more than a 45-day gap in coverage. Exclusion criteria included those being treated for a cancer diagnosis, or in long-term residential care or hospice care, those dually eligible for Medicaid and Medicare, or receiving only limited benefits and those having a residential zip code outside of Illinois. Criteria for determining no, possible, and probable misuse are based on number of prescriptions for opioids received, and the number of different pharmacies where prescriptions were filled as well as morphine millgram equivalents per day [33]. Persons with an OUD were identified through analysis of claims data indicating receipt of treatment services for an OUD during the year, as specified in the SUD Technical Specifications document [34]. All figures are percentages unless otherwise indicated. Significance levels for all categorical variables are based on the Pearson Chi-square statistic while effect size is based on Cramer's V. The significance level for age in years is based on a bivariate regression model estimated with robust standard errors. Effect size for the regression model is based on the R-squared result. (2) ***: *p* < 0.001; NS: Non-Significant. (3) Cramer's V Interpretation: 0.0: no association; 0.1–0.3: weak association; 0.4–0.5: medium association; > 0.5: strong association.

**Table 2.** Co-occurring substance use and mental health disorders by opioid use category.

**Project**	**Prescription opioid misuse category**							
	**No detected opioid use (*N* = 954903)**	**No misuse (*N* = 82814)**	**Possible misuse (*N* = 36795)**	**Probable misuse (*N* = 4644)**	**Opioid use disorder (*N* = 23323)**	**Total (*N* = 1102479)**	**Sig**	**Cramer's V/R-Squared**
**Substance use disorders**								
Alcohol (%)	1.7	2.6	5.1	8.1	18.7	2.3	***	0.17
Cannabis (%)	1.2	1.7	3.0	6.1	11.7	1.5	***	0.13
Cocaine (%)	0.4	0.6	1.4	4.0	15.7	0.8	***	0.27
Other stimulants (%)	0.2	0.3	0.5	1.0	3.7	0.3	***	0.90
Sedatives (%)	0.1	0.1	0.3	1.2	4.0	0.2	***	0.14
Tobacco (%)	4.5	10.1	21.7	31.8	40.4	6.3	***	0.26
Hallucinogens (%)	0.0	0.0	0.1	0.3	0.9	0.1	***	0.05
Inhalants (%)	0.0	0.0	0.0	0.0	0.1	0.0	***	0.01
Number of Co-occurring SUDs (*mean*/*SD*)	0.1 (0.5)	0.2 (0.5)	0.3 (0.7)	0.6 (0.9)	1.1 (1.4)	0.1 (0.5)	***	0.11
**Mental health disorders**								
Anxiety (%)	7.3	11.6	29.4	42.4	34.3	9.1	***	0.21
Depression (%)	6.2	8.7	23.5	33.6	32.8	7.7	***	0.19
Bipolar (%)	2.9	3.3	10.5	18.8	19.0	3.6	***	0.15
Schizophrenia/psychotic disorder (%)	2.6	1.6	4.3	8.3	11.2	2.8	***	0.08
Post-traumatic stress disorder (%)	0.9	1.4	4.3	7.9	6.1	1.2	***	0.09
Personality disorder (%)	0.6	0.7	2.2	4.1	3.7	0.7	***	0.67
ADHD (%)	0.6	0.6	1.4	3.6	2.7	0.7	***	0.05
Autism (%)	0.2	0.1	0.1	0.2	0.2	0.2	***	0.01
Intellectual disability (%)	0.5	0.2	0.4	0.7	0.4	0.5	***	0.01
Number of Co-occurring MHDs (mean/*SD*)	0.2 (0.6)	0.3 (0.7)	0.8 (1.1)	1.2 (1.3)	1.1 (1.4)	0.3 (0.7)	***	0.07

Note: (1) Results based on all adult Illinois residents ages 18 to 64 years of age who were enrolled in a Medicaid/CHIP managed care program in calendar year 2018 for 300 or more days with no more than a 45-day gap in coverage. Exclusion criteria included those being treated for a cancer diagnosis, or in long-term residential care or hospice care, those dually eligible for Medicaid and Medicare, or receiving only limited benefits and those having a residential zip code outside of Illinois. Criteria for determining no, possible, and probable misuse are based on number of prescriptions for opioids received, and the number of different pharmacies where prescriptions were filled as well as morphine millgram equivalents per day [33]. Persons with an OUD were identified through analysis of claims data indicating receipt of treatment services for an OUD during the year, as specified in the SUD Technical Specifications document [34]. Significance levels for all categorical variables are based on the Pearson Chi-square statistic while effect size is based on Cramer's V. Signficance for the comparison of mean numbers of substance use and mental health disorders by potential misuse status are based on bivariate regressions estimated with robust standard errors. Effect sizes for the regression models are based on the R-squared result. In these models, OUD was excluded from the calculation of number of co-occurring SUDs. (2) ***: p < 0.001. (3) **Cramer's V Interpretation:** 0.1–0.3: weak association; 0.4–0.5: medium association; > 0.5: strong association.

### 4.4. Multinomial logistic regressions

Multinomial logistic regression results for co-occurring SUDs and mental health disorders by opioid use category are shown in Table 3. Overall, the obtained relative risk ratios follow the same general pattern as the bivariate analyses, whereby beneficiaries with an OUD or with probable prescription misuse had the highest risk for most substances and mental health disorders, followed by those in the possible misuse group and then by those in the no detected prescription opioid misuse group. With a few exceptions (e.g., personality disorder and ADHD for the no detected prescription opioid misuse group; autism and intellectual disability for both the possible and probably prescription opioid misuse groups; and intellectual disability of the OUD group), all comparisons were statistically significant. Among the more prominent results, those with an OUD were at an especially high relative risk for a number of SUDs: sedative use disorder {relative risk ratio (rrr) = 63.5, 95% *CI* = [56.59, 71.23]}; cocaine use disorder [rrr = 32.4, 95% *CI* = (30.81, 34.15)]; and other stimulants {rrr = 16.7, 95% *CI* = [16.7, (15.25, 18.19)]}. Among this group, while still elevated relative to those with no opioid use [rrr = 11.6, 95% *CI* = (11.29, 12.00)], Tobacco use disorder was only the fifth most prominent co-occurring SUD among those with an OUD. Sedative use disorder was also at an elevated risk among those with probable prescription opioid misuse [rrr = 18.6, (13.75, 25.21)], though not nearly to the same degree as among those with an OUD.

The results were more mixed for mental health disorders. Persons with an OUD or with probable prescription opioid misuse continued to have the highest risk for most of the co-occurring health conditions, though the differences between these two groups were much less pronounced than for co-occurring SUDs. Among the higher risk conditions for both groups were anxiety disorders, PTSD, depression, ADHD, and bipolar disorder. Similar to the bivariate results, persons in the probable prescription opioid misuse category had higher risks for a number of the assessed mental health disorders including anxiety disorder [rrr = 8.6, 95% *CI* = (8.08, 9.21)]; PTSD [rrr = 8.1, (95% *CI* = 7.2,9.1)]; and ADHD [rrr = 7.9, 95% *CI* = (6.57, 9.22)].

## 5. Discussion and conclusions

To specifically address the main study motivation of examining the co-occurrence of behavioral health conditions among Medicaid beneficiaries misusing prescription opioids, the findings unequivocally indicate those with probable prescription opioid misuse are at high risk for co-occurring SUDS and mental health disorders. While beneficiaries with an OUD appear to be at highest risk for a co-occurring SUD — particularly sedatives, tobacco, cocaine, and alcohol—those with probable prescription opioid misuse, while also at risk for a co-occurring SUD, have an equal or sometimes higher risk for a co-occurring mental health disorder compared with persons with an OUD. The most common co-occurring mental health conditions among these two groups misusing or dependent on opioids were as follows: anxiety, PTSD, depression, bipolar disorder, and ADHD. Moreover, we should note that persons assessed as possibly misusing prescription opioids were also at higher risk for SUDs and mental health disorders, though to a lesser degree than those with probable prescription opioid misuse or an OUD.

**Table 3.** Multinomial logistic regression by opioid use category.

**Project**	**Prescription opioid misuse category**											
	**No misuse**			**Possible misuse**			**Probable misuse**			**Opioid use disorder**		
	**rrr**	**95% *CI***	Sig	**rrr**	**95% *CI***	Sig	**rrr**	**95% *CI***	Sig	**rrr**	**95% *CI***	Sig
**Substance use disorders**												
Alcohol	1.5	[1.41, 1.56]	***	2.9	[2.75, 3.06]	***	4.4	[3.89, 4.91]	***	9.6	[9.23, 10.01]	***
Cannabis	1.5	[1.43, 1.61]	***	3.2	[3.00, 3.43]	***	6.6	[5.76, 7.49]	***	10.5	[10.01, 11.03]	***
Cocaine	1.3	[1.15, 1.41]	***	3.2	[2.85, 3.49]	***	8.3	[7.00, 9.73]	***	32.4	[30.81, 34.15]	***
Other stimulants	1.3	[1.11, 1.46]	**	2.1	[1.81, 2.52]	***	4.6	[3.35, 6.31]	***	16.7	[15.25, 18.19]	***
Sedatives	1.0	[0.77, 1.39]	***	4.7	[3.78, 5.87]	***	18.6	[13.75, 25.21]	***	63.5	[56.59, 71.23]	***
Tobacco	2.1	[2.08, 2.19]	***	4.8	[4.68, 4.95]	***	7.8	[7.26, 8.32]	***	11.6	[11.29, 12.00]	***
Hallucinogens	0.9	[0.64, 1.36]	NS	2.6	[1.78, 3.85]	***	8.7	[5.00, 15.19]	***	15.9	[13.3, 19.0]	***
Inhalants	0.7	[0.22, 2.32]	NS	1.5	[0.46, 4.81]	NS	3.5	[0.48, 25.6]	NS	9.3	[5.23, 16.37]	***
**Mental health disorders**												
Anxiety	1.5	[1.45, 1.53]	***	4.5	[4.41, 4.64]	***	8.6	[8.08, 9.21]	***	6.8	[6.61, 7.05]	***
Depression	1.3	[1.24, 1.31]	***	3.8	[3.65, 3.87]	***	6.3	[5.92, 6.77)	***	6.7	[6.54, 6.96]	***
Bipolar	1.0	[0.96, 1.05]	NS	3.3	[3.20, 3.54]	***	6.6	[6.12, 7.19]	***	6.7	[6.43, 6.93]	***
Schizophrenia/psychotic disorder	0.6	[0.58, 0.65]	***	1.5	[1.44, 1.61]	***	2.9	[2.60, 3.25]	***	3.3	[3.18, 3.49]	***
Post-traumatic stress disorder	1.3	[1.23, 1.41]	***	4.2	[3.93, 4.43]	***	8.1	[7.20, 9.10]	***	6.9	[6.52, 7.39]	***
Personality disorder	1.0	[0.95, 1.14]	NS	3.2	[2.96, 3.48]	***	6.5	[5.58. 7.60]	***	5.9	[5.48, 6.41]	***
ADHD	1.0	[0.95, 1.15]	NS	2.7	[2.46, 3.00]	***	7.9	[6.57, 9.22]	***	5.0	[4.58, 5.49]	***
Autism	0.3	[0.20, 0.39]	***	0.8	[0.58, 1.16]	NS	1.6	[0.81, 3.26]	NS	0.8	[0.56, 1.12]	NS
Intellectual disability	0.3	[0.24, 0.35]	***	0.9	[0.73, 1.02]	NS	1.3	[0.87, 1.87]	NS	0.7	[0.54, 0.82]	***

Note: (1) Results based on all adult Illinois residents ages 18 to 64 years of age who were enrolled in a Medicaid/CHIP managed care program in calendar year 2018 for 300 or more days with no more than a 45-day gap in coverage. Exclusion criteria included those being treated for a cancer diagnosis, or in long-term residential care or hospice care, those dually eligible for Medicaid and Medicare, or receiving only limited benefits and those having a residential zip code outside of Illinois. Criteria for determining no, possible, and probable misuse are based on number of prescriptions for opioids received, and the number of different pharmacies where prescriptions were filled as well as morphine millgram equivalents per day [33]. Persons with an OUD were identified through analysis of claims data indicating receipt of treatment services for an OUD during the year, as specified in the SUD Technical Specifications document [34]. The reference category for all multinomial logistic models was Medicaid beneficiaries with no detected opioid use in the past year. Each model controlled for the following covariate effects: gender, race/ethnicity, age in years, marital status. veteran status, and citizenship. (2) rrr: relative risk ratio. (3) **: *p* < 0.01; ***: *p* < 0.001; NS: Non-Significant.

Although other studies have indicated a pathway from prescription opioid misuse to OUD (e.g., [44]), based on our data and analyses, we cannot determine if this is the case in this sample of Medicaid beneficiaries. Such a determination would require longitudinal data; prior information for those with an OUD and follow-up information for those with probable or possible opioid misuse. However, it does seem likely that some non-trivial proportion of those misusing prescription opioids in 2018 when the study data were collected have since developed and/or been treated for an OUD. Research using multiple years of Medicaid data might be able to assess the extent to which this transition occurs among the probable and possible prescription opioid misuse groups.

Lacking longitudinal data, we might speculate as to why polypharmacy is especially prominent among persons with an OUD but not as prevalent among those misusing prescription opioids, whereas mental health conditions appear to be near equally prevalent among both groups. One hypothesis is that co-occurring mental health disorders constitute an important risk factor for developing an OUD among the probable prescription opioid misuse group; those already evidencing these conditions could be at most risk for progressing to an OUD. On the other hand, polypharmacy, as evidenced by the highest rates of and risk for co-occurring SUDs among persons with an OUD, could develop alongside more frequent use of and ultimately dependence on opioids. That is, co-occurring SUDs are not risk factors for developing an OUD to the same extent as mental health disorders. Instead, we hypothesize that persons in the probable prescription opioid misuse group who progress to an OUD and whose rates of co-occurring SUDs were below those of persons with an OUD at the time the data were collected, will eventually show an increased prevalence of SUDs matching those of persons in the OUD group. In other words, mental health disorders might be determinative as a risk factor for opioid prescription misuse and an OUD whereas SUDs, while perhaps partially determinative as a risk factor for progressively increasing opioid use, might be correlated with increased opioid use that develops synchronously over time.

Although not a focus of the study, we also want to highlight several findings that emerged from the analyses, the first being gender differences in the prevalence of prescription opioid misuse and the second being the high prevalence of a co-occurring tobacco use disorder among persons with an OUD. We found a higher prevalence of prescription opioid misuse among women compared with men, but a higher prevalence of OUD among men compared with women. This finding is consistent with prior research that shows that women are more likely to use and misuse prescription opioids than men. [45],[46] Reasons for this discrepancy include women having higher rates of conditions such as chronic pain that are treated with opioids and higher rates of doctor visits, an important predictor of prescription opioid use and misuse [46]. As this was a secondary analysis of claims data, we do not have additional information to explain the higher prevalence of prescription opioid misuse among women in our sample. Clearly, however, more research is needed to better understand the reasons underlying these gender differences found in our and other studies of prescription opioid misuse.

The prevalence of a co-occurring tobacco use disorder (40.2%) among persons with an OUD and, to a lesser extent but still higher than the general population prevalence of tobacco use (13.7%), those with probable (31.8%) and possible (21.7%) prescription opioid misuse also had an elevated prevalence of tobacco use disorder [47]. Nicotine and opioids share a number of neural pathways that mediate their effects and they appear to act synergistically to potentiate continued and increasing use of both substances [48]. Especially important in the context of this study and the use of prescription opioid analgesics, chronic nicotine use might heighten the experience of pain as well as increase tolerance to pain medication, requiring higher dosages of pain medication to achieve pain relief among tobacco users in comparison to non-tobacco users [49]. One theory is that tobacco use disorder might be a risk factor for progression from opioid use to an OUD. Research also shows that co-treatment of tobacco use and OUD can lead to a higher chance of abstinence and a longer period of recovery from opioid use [48]. From a treatment standpoint then, programs that address tobacco and opioid use when they co-occur might prove more effective than treating one or the other disorder although clear best-practice treatments for smoking cessation by persons with an OUD have yet to be determined [49].

During research for the Illinois SUPPORT project mentioned in the Introduction [6], we found that despite considerable and successful efforts by the state to increase the number of health care providers licensed to prescribe buprenorphine as a treatment for an opioid addiction, many were not prescribing at all or were prescribing well below their licensed limits. A survey of these providers found that patient complexity and lack of available community-based behavioral health treatment supports were among the main reasons they limited their practices. (See also, [50],[51]).

The results of this study indicate these provider concerns are well-founded. Among adult Medicaid beneficiaries, a person seeking medication treatment for their OUD is highly likely to present with multiple SUDS and mental health disorders, which also require medical attention and coordinated behavioral health care interventions. These results are consistent with other research that has found persons with a mental illness are more likely to use prescription opioids. Based on U.S. national data, sixteen percent of Americans with a mental illness receive over half of all prescribed opioids [28],[32], thought to reflect the higher rates of pain in the population with mental illnesses as well as the antidepressant effects of opioids.

It is likely the case that many general practice physicians or other community-based health care providers are not well equipped to either treat or refer such patients for other needed services, whether owing to a lack of in-office referral systems and/or a lack of proximal community-based behavioral health care treatment programs. Developing more comprehensive systems of care built around providing medication-assisted treatment might do more to promote such treatment than will recruiting and training more providers to prescribe buprenorphine. We note the recent NIH-funded Helping to End Addiction Long-term Initiative (NIH HEAL), that is developing and studying a collaborative care model designed specifically to address the known high rates of co-occurring mental illness and OUD [52].

## 6. Limitations

We have mentioned that the lack of longitudinal data precluded us from assessing causal effects, limiting the findings to associative. More research is needed to determine the extent to which persons with co-occurring mental health and SUDS, particularly tobacco use disorder, are at higher risk to progress into opioid misuse and ultimately an OUD. A second important limitation is that the Medicaid data analyzed were restricted to Illinois. Therefore, the findings might not generalize to Medicaid populations in other states or to non-Medicaid populations. Medicaid data have their own limitations in that they are derived from claims information, filed when a beneficiary receives treatment services for various health conditions for which the state is seeking federal reimbursement. Beneficiaries who do not seek treatment for but who have a specific condition such as OUD, will not be identified as such. Hence, the prevalence of OUD, other SUDs, and mental health disorders in our sample are very likely lower bound estimates.

## Use of AI tools declaration

The authors declare they have not used Artificial Intelligence (AI) tools in the creation of this article.
