# Supplementary material for: Mental health and substance use disorder comorbidities among Medicaid beneficiaries: Associations with opioid use disorder and prescription opioid misuse
Source: AIMS Public Health. 2023 Aug 10;10(3):658–77. doi: 10.3934/publichealth.2023046 (PMC10567978; doi:10.3934/publichealth.2023046)
Supplement: Supplementary file 1 [file publichealth-10-03-046-s001.pdf]

Public Health

AIMS Public Health, 10(3): 658–677.
DOI: 10.3934/publichealth.2023046
Received: 23 March 2023
Revised: 10 July 2023
Accepted: 25 July 2023
Published: 10 August 2023

http://www.aimspress.com/journal/aimsph

Research article

Mental health and substance use disorder comorbidities among Medicaid beneficiaries: Associations with opioid use disorder and prescription opioid misuse

James A. Swartz[1,*], Dana Franceschini[1] and Kamryn Scamperle[2]

[1] Jane Addams College of Social Work, University of Illinois Chicago
[2] University of Texas Health Science Center at Houston

* **Correspondence:** Email: jaswartz@uic.edu; Tel: +3129968560.

Supplementary

Table S1. Imputed multinomial logistic regression results by opioid use category.

Project	Prescription Opioid Misuse Category											
	No Misuse			Possible Misuse			Probable Misuse			Opioid Use Disorder		
	rrr	95% CI	Sig	rrr	95% CI	Sig	rrr	95% CI	Sig	rrr	95% CI	Sig
**Substance use disorders**												
**Alcohol**	1.5	[1.42, 1.56]	***	2.9	[2.77, 3.06]	***	4.5	[4.04, 5.02]	***	9.5	[9.13, 9.84]	***
**Cannabis**	1.5	[1.43, 1.61]	***	3.3	[3.01, 3.48]	***	6.7	[5.93, 7.59]	***	10.6	[10.15, 11.12]	***
**Cocaine**	1.3	[1.17, 1.42]	***	3.1	[2.85, 3.44]	***	8.7	[7.44, 10.09]	***	32.4	[30.76, 33.88]	***
**Other stimulants**	1.3	[1.12, 1.52]	**	2.2	[1.88, 2.59]	***	5.0	[3.74, 6.76]	***	17.2	[15.85, 18.75]	***
**Sedatives**	1.1	[0.82, 1.42]	***	4.7	[3.83, 5.84]	***	20.2	[15.25, 26.74]	***	66.8	[59.93, 74.41]	***
**Tobacco**	2.2	[2.09, 2.20]	***	4.9	[4.77, 5.03]	***	8.1	[7.60, 8.63]	***	11.7	[11.39, 12.06]	***
**Hallucinogens**	1.0	[0.71, 1.42]	NS	2.6	[1.82, 3.80]	***	8.6	[5.04, 14.71]	***	16.5	[13.93, 19.56]	***
**Inhalants**	0.7	[0.22, 2.22]	NS	1.4	[0.45, 4.68]	NS	3.4	[0.47, 25.1]	NS	9.2	[5.29, 16.08]	***
**Mental health disorders**												
**Anxiety**	1.5	[1.46, 1.53]	***	4.6	[4.47, 4.70]	***	8.7	[8.21, 9.28]	***	6.9	[6.67, 7.07]	***
**Depression**	1.3	[1.26, 1.32]	***	3.8	[3.71, 3.91]	***	6.4	[6.00, 6.80)	***	6.8	[6.57, 6.97]	***
**Bipolar**	1.1	[0.99, 1.07]	NS	3.4	[3.30, 3.54]	***	6.8	[6.27, 7.29]	***	6.8	[6.57, 7.06]	***
**Schizophrenia/psychotic disorder**	0.6	[0.59, 0.66]	***	1.5	[1.49, 1.65]	***	2.9	[2.65, 3.23]	***	3.3	[3.19, 3.49]	***
**Post-traumatic stress disorder**	1.3	[1.26, 1.43]	***	4.2	[3.94, 4.40]	***	8.1	[7.30, 9.10]	***	6.9	[6.52, 7.33]	***
**Personality disorder**	1.1	[0.97, 1.15]	NS	3.3	[3.03, 3.53]	***	6.4	[5.51. 7.42]	***	6.1	[5.64, 6.55]	***
**ADHD**	1.1	[0.97, 1.16]	NS	2.8	[2.59, 3.11]	***	7.8	[6.65, 9.16]	***	5.0	[4.57, 5.42]	***
**Autism**	0.3	[0.21, 0.39]	***	0.8	[0.60, 1.15]	NS	1.6	[0.84, 3.13]	NS	0.8	[0.60, 1.12]	NS
**Intellectual disability**	0.3	[0.25, 0.36]	***	0.9	[0.76, 1.05]	NS	1.4	[0.96, 1.96]	NS	0.6	[0.52, 0.78]	***

Note: (1) Results based on all adult Illinois residents ages 18 to 64 years of age enrolled in a Medicaid/CHIP managed care program in calendar year 2018 for 300 or more days with no more than a 45-day gap in coverage. Exclusion criteria included: those being treated for a cancer diagnosis, or in long-term residential care or hospice care; those dually eligible for Medicaid and Medicare, or receiving only limited benefits; and those having a residential zip code outside of Illinois. Criteria for determining no, possible, and probable misuse are based on the number of prescriptions for opioids received, and the number of different pharmacies where prescriptions were filled as well as morphine millgram equivalents per day [33]. Persons with an OUD were identified through analysis of claims data indicating receipt of treatment services for an OUD during the year, as specified in the SUD Technical Specifications document [34]. The reference category for all multinomial logistic models was Medicaid beneficiaries with no detected opioid use in the past year. Each model controlled for the following covariate effects: gender, race/ethnicity, age in years, marital status. veteran status, and citizenship. Regressions were run following multiple imputation through chained equations with information for marital status and race/ethnicity imputed over 15 replications. (2) **: $p < 0.01$; ***: $p < 0.001$; NS: Non-Significant

© 2023 the Author(s), licensee AIMS Press. This is an open access article distributed under the terms of the Creative Commons Attribution License (http://creativecommons.org/licenses/by/4.0)
